# Effect of Vitamin C Source on Its Stability during Storage and the Properties of Milk Fermented by *Lactobacillus rhamnosus*

**DOI:** 10.3390/molecules26206187

**Published:** 2021-10-14

**Authors:** Agata Znamirowska, Katarzyna Szajnar, Małgorzata Pawlos

**Affiliations:** Department of Dairy Technology, Institute of Food Technology and Nutrition, College of Natural Sciences, University of Rzeszow, Cwiklińskiej 2D, 35-601 Rzeszów, Poland; aznamirowska@ur.edu.pl (A.Z.); mpawlos@ur.edu.pl (M.P.)

**Keywords:** fermented milk, vitamin C, *Lactobacillus rhamnosus*, probiotic bacteria

## Abstract

The enrichment of commonly consumed foods with bioactive components might be helpful in promoting health and reducing the risk of disease, so the enrichment of probiotic fermented milk with vitamin C can be considered appropriate. The effect of vitamin C addition depends on the source of origin (rosehip, acerola and ascorbic acid in powder form) on the growth and survival of *Lactobacillus rhamnosus* and the quality of fermented milk on the 1st and 21st day of storage was analyzed. The pH, total acidity, vitamin C, syneresis, color, texture profile and numbers of bacterial cells in fermented milk were determined. The organoleptic evaluation was also performed. The degradation of vitamin C in milk was shown to depend on its source. The lowest reduction of vitamin C was determined in milk with rosehip. The least stable was vitamin C naturally found in control milk. The addition of rosehip and acerola decreased syneresis and lightness of milk color, increasing the yellow and red color proportion. In contrast, milk with ascorbic acid was the lightest during the whole experimental period and was characterized by a very soft gel. The growth of *Lactobacillus rhamnosus* during fermentation was most positively affected by the addition of rosehip. However, the best survival of *Lactobacillus rhamnosus* was demonstrated in milk with acerola. On the 21st day of storage, the number of *L. rhamnosus* cells in the control milk and the milk with vitamin C was >8 log cfu g^−1^, so these milks met the criterion of therapeutic minimum. According to the assessors, the taste and odor contributed by the addition of rosehip was the most intense of all the vitamin C sources used in the study.

## 1. Introduction

The consumption of probiotic foods is steadily increasing, and consumers are choosing these products especially for their taste and beneficial health aspects. It is assumed that probiotic bacteria have the potential to provide health benefits that include intestinal microbial balance and mineral absorption, prevention of constipation, reduction of cholesterol levels in serum, and improvements in lactose intolerance and blood pressure [1,2,3].

*Lactobacillus rhamnosus* (LGG) with probiotic properties is a promising natural alternative to commercial additives in the food industry [4]. This bacteria exhibits most of the characteristics generally desirable for a good probiotic strain, including the ability to survive passage through the human gastrointestinal tract after ingestion and the capacity to temporarily colonize the ileum and colon. The beneficial health properties of LGG depend partially on its prolonged residence in the gastrointestinal tract and strong adhesion ability. Therefore, LGG is an effective starter for fermented milk production, while fermented milk with LGG has various probiotic functions for adults and children. Moreover, this bacilli has the benefits of being resistant to low pH levels and has been reported to show excellent viability [5]. Furthermore, it is a potential candidate for the synthesis of acetoin and diacetyl, which are butter-like flavor compounds widely used in the dairy industry [6].

However, it is crucial to ensure an adequate number of viable bacterial cells, which is the “therapeutic minimum” that should be regularly consumed to achieve the “probiotic” effect for the consumer. According to the International Dairy Federation Recommendation, probiotic products should contain at least seven log CFU g^−1^ of lactic acid bacteria. Therefore, the survival of these bacteria during the shelf-life and until consumption is an important aspect. Furthermore, criteria for the selection of probiotic strains include safety as well as technological, sensory and functional properties of the strains [7,8].

The enrichment of commonly consumed foods with bioactive components may help promote health and reduce disease risk, so the enrichment of probiotic fermented milk with vitamin C may be considered appropriate.

Due to the very good solubility of vitamin C and active transport, it is absorbed by the body in about 70–80% (from a dose of 180 mg in non-smokers) [9,10]. The main organs where the absorption process occurs are the duodenum and the proximal part of the small intestine [10,11]. The efficiency of this process strongly depends on the condition of the body. It might be impaired by vomiting, lack of appetite, digestion and absorption disorders, intestinal dysfunction, smoking and use of some drugs (e.g., aspirin) [9,11].

Vitamin C is the most well-known antioxidant. With its antioxidant properties, this vitamin provides a protective role in cardiovascular disease. Zhang et al. [12], in a study conducted on cigarette smokers, demonstrated that ascorbic acid in conjunction with other antioxidants (including vitamin E) inhibited elevated markers of lipid peroxidation induced by smoking in smokers. Antioxidants, including vitamin C, were confirmed to have a protective role in coronary heart disease and cardiovascular disease. Based on its antioxidant capacity, ascorbic acid protects body cells from oxidative stress [12].

Acerola (*Malpighia emarginata* DC.) is one of the richest natural sources of ascorbic acid in the world. It is grown in areas from southern Texas, Mexico, and Central America to northern South America and the Caribbean. It is recently introduced to subtropical areas around the world, including India. The fruit is consumed as fresh juice and as cherry consumed daily by many native tribes, or by animals [13]. Acerola is a good source of vitamin C, which also contains amino acids, phenolic compounds, and carotenoids, classifying it as a nutraceutical [14,15,16]. The vitamin C content of acerola fruit varies depending on ripening stages, cultivar, growing location, and environmental factors [15,17,18]. Vitamin C content is high in unripe fruit, about 1.9% of juice and decreases during ripening to about 0.97% of juice in ripe fruit [17].

Rosehips are wild pseudo fruits derived from plants in the genus *Rosa*, widely cultivated in Europe, the Middle East, Asia, and North America. The most abundant and most studied species in Europe is *Rosa canina*, which is a native shrub. This fruit is usually consumed as tea, jelly, jam and beverages, and is now also used as an ingredient in probiotic drinks, yoghurts and soups [19]. Rose hips are also rich in vitamin C. The content of L-ascorbic acid in these fruits ranges from 0.5 to 2.0%. Moreover, rose hips contain vitamins, e.g., B1, B2, PP, A, E, K, carotenoids, and polyphenolic compounds [20].

It was shown that the stability of ascorbic acid in rosehip fruit is higher in the fruit matrix than in the extracts. Furthermore, flavonoids from rosehips could prevent the oxidation of ascorbic acid [21,22].

The bioavailability of water-soluble vitamins in the gastrointestinal tract, including the oral cavity, stomach, and small intestine, could vary depending on pH, temperature, binding to polypeptides and polysaccharides and the presence of metal ions and digestive enzyme inhibitors [23].

Depending on the additional compounds present in acerola and rosehip and the resulting stability of vitamin C in beverages, a study was conducted on three different sources of vitamin C: rosehip, acerola and ascorbic acid. Therefore, the effect of vitamin C addition, in dependence on the source, on the growth and survival of *Lactobacillus rhamnosus* and the quality of fermented milk on the 1st and 21st day of storage was analyzed.

## 2. Results and Discussion

### 2.1. Physicochemical Properties of Fermented Milk

#### The pH Value and Total Acidity

The milk was enriched with vitamin C at a dose of approximately 30 ± 3 mg 100 g^−1^ in the form of rosehip (DR), acerola (AC) and ascorbic acid (VC). As expected, the pH value of DR, AC and VC milk with vitamin C addition was significantly lower before fermentation compared to the control milk (Table 1). Before fermentation, the addition of rosehip to DR milk decreased the pH value the most, whereas after fermentation, the pH was the highest in DR milk with rosehip and VC with ascorbic acid, which was also associated with the lowest lactic acid content in this milk. On the 1st and 21st day of storage, the highest amount of lactic acid was determined in AC milk with acerola. Lactic acid content did not change significantly during refrigerated storage of milk, which is also confirmed by a two-way analysis of variance (Table 2).

In Linares et al. [24] study, *L.* *rhamnosus* was suspected of assimilating L-ascorbate, and in vitro experiments were performed to evaluate the putative catabolism. The results showed a global increase in the maximum absorbance obtained in the presence of D-glucose or L-ascorbate. There was a correlation with the presence of carbohydrates, as no increase in absorbance was observed in the negative control (no carbohydrate). However, control abiotic tests (performed without cell suspension) showed that this phenomenon was partly due to the yellow coloration of the substrate observed in the presence of l-ascorbate. In the study of Linares et al. [24], the growth of *l. rhamnosus* GG was supported by l-ascorbate because it was the only amino acid compound that could be used as a carbon source. l-ascorbate might serve as a reducing agent or a cofactor to promote utilising other carbon sources present in the medium (for example, amino acids). The main hypothesis explaining the presence of 12C-lactate is the catabolism of glucogenic amino acids. The biotransformation of 12C-amino acids could generate 12C-pyruvate and consequently 12C-lactate through lactate dehydrogenase activity. This pathway could also lead to the production of 12C-acetate. According to the metabolism of 13C-ascorbate, 12C-lactate might be formed only by amino acid catabolism. After 67 h of growth, the experimental results clearly showed that when bacteria assimilated amino acids, the kinetics of lactate production showed a much higher increase than the kinetics of acetate, which increased more or less consistently. Amino acid degradation generates more lactate than acetate (about three times more), while the opposite is true for ascorbate-only degradation. Acerola is rich in vitamin C, pectin, pectolytic enzymes, carotenoids and fiber, and vitamins and bioactive substances such as thiamine, riboflavin, niacin, proteins and mineral salts, mainly iron, calcium and phosphorus. The amino acid composition of some varieties of acerola (Vietnam) was notable, with an exceptionally high concentration of proline (858 mg kg^−1^), accounting for almost half of the total amino acid content (1733 mg kg^−1^). These fruits also contained high levels of phenylpropanoids, representing 30% of the total polyphenol content [15]. This fact might be explained by the high total acidity expressed as lactic acid in AC milk compared to DR and VC milk.

### 2.2. The Vitamin C

Before fermentation, 10.5 mg 100 g^−1^ of vitamin C was determined in control milk, whereas in milk with added vitamin C from different sources, the vitamin C content was 43 mg 100 g^−1^. After fermentation, the vitamin C content decreased significantly in all milk groups, even in control milk, compared to the amount of vitamin C before fermentation. Fermentation by *Lactobacillus rhamnosus* of control milk K and rosehip DR, and acerola AC decreased the vitamin C content by about 1 mg 100 g^−1^. In comparison, in VC milk with ascorbic acid, it only reduced by 0.7 mg 100 g^−1^.

Our study did not show high vitamin C degradation, and there are reports that the acidic environment stabilizes vitamin C. According to Pérez-Vicente et al. [25], loss of vitamin C in pomegranate juice occurred due to pH changes. Yuan and Chen [26] studied the distribution of vitamin C in aqueous solutions. They demonstrated that low pH conditions promoted the formation of furfural, 2-furoesic acid and 3-hydroxy-2-pyrone. Some authors [27] indicate that a specific increase in the vitamin C content of fermented dairy products might be related to its synthesis by selected lactic acid bacteria. Ascorbic acid as an antioxidant reduces the number of substances formed during microbial metabolism [28]. In this study, extending the storage time of fermented milk to 21 days resulted in a continued decrease in vitamin C content from 4.4 mg 100 g^−1^ in control milk to 1.5 mg 100 g^−1^ in DR milk compared to the amount of vitamin C on the first day of storage. This implies that the least stable vitamin C was in the control milk, where its loss was 51.8% compared to its content before milk fermentation.

Differences in vitamin C content according to the source of origin were also determined. In DR milk with rosehip, the reduction of vitamin C content during storage was the lowest (5.9%). In VC milk with ascorbic acid, the degree of vitamin C degradation was the highest and amounted to 8.0% concerning its content before milk fermentation.

The least reduction of vitamin C in DR milk during storage might be related to the rosehip specific antioxidants. Phenolic compounds, including tannins, flavonoids, phenolic acids and anthocyanins, are an essential group of biologically active components in rosehip fruits [29]. Phenols are well known for their antioxidant properties, and several studies investigate the content and composition of polyphenols in different rose species. However, various quantitative and qualitative reports of the phenolic profile of roses are found in the literature. The main flavonoids are methyl gallate, catechin [30,31], epicatechin [31,32,33], rutin, eriocitrin, quercetin, apigenin-7-*O*-glucoside, kaempferol, [30,31], quercetin and quinic acid [32]. Tumbas et al. [33] and Hosni et al. [34] identified quercetin and ellagic acid. In contrast, Türkben et al. [35] and Olsson et al. [36] found quercetin and catechin to be the essential phenolic components in rose fruits with the absence of ellagic acid and kaempferol. Demir et al. [30] and Elmastas et al. [31] found phenolic acids in rosehip fruits, including gallic acid, 4-hydroxybenzoic acid, caftaric acid, 2,5-dihydroxybenzoic acid, chlorogenic acid, t-caffeic acid, p-coumaric acid and ferulic acid. Nadpal et al. [32] reported protocatechuic acid except for the previously mentioned compounds.

Ilic and Ashoor [37] indicate that raspberry-infused yoghurt contained more reductants which decreased the rate of vitamin C degradation during storage.

The loss of vitamin C content in milk and dairy products during storage is affected by light, oxygen, iron, copper; moreover, it depends on the type of packaging [38]. FAO/WHO recommends 45–70 mg/day RNI (Recommended Nutrient Intakes) for vitamin C for an adult. Considering the vitamin C content in milk in our study, consumption of 100–150 mL of DR, VC and AC milk would meet the daily requirement for vitamin C [39].

### 2.3. Syneresis

The results of syneresis of milk fermented by *L. rhamnosus* are shown in Table 1. Both control milk and milk with added vitamin C from different sources (DR, AC, VC) characterized high syneresis. After fermentation, the highest syneresis was determined in AC milk, where the pH value was also the lowest and the lactic acid content the highest. Many authors explain that the ability to hold water in an acid gel depends on the acidity of the milk [40,41,42].

The lowest level of syneresis after fermentation was found in VC milk (61.37%) and DR milk (61.93%), in which the highest pH value was determined. An enhancement of syneresis with increasing storage time was found in the control milk and the milk with ascorbic acid (VC). However, in milk with rosehip (DR) and acerola (AC), an increase in storage time from 1 to 21 days resulted in a decrease in separated whey. The two-way analysis of variance indicated that the syneresis of fermented milk depended on the storage time, source of vitamin C, and these factors’ interaction (Table 2). According to Sun et al. [43], milk fermented by *L. rhamnosus* showed a water retention capacity ranging from 34.7–39.3% calculated as a percentage of remaining sediment after centrifugation. In our study, syneresis was given as a percentage of whey separated, hence relating the results to the study of Sun et al. [43] could be assumed to be similar. Furthermore, these authors showed that nutrient enrichment of milk contributes to water retention capacity. Concerning our study, the addition of rosehip and ascorbic acid improved the water retention capacity of milk fermented by *L. rhamnosus.*

### 2.4. Color

The color components of milk fermented by *L. rhamnosus* depended on the source of added vitamin C. The lightness of L* was most intensely reduced by the addition of rosehip due to carotenoids, polyphenolic compounds such as tannins, flavonoids and anthocyanins in rosehip [44] (Table 1). Reduction in color lightness was also caused by the addition of acerola, which also contains phenolic compounds including anthocyanins and flavonoids and carotenoids [45]. In this study, the addition of ascorbic acid did not reduce color lightness on the first day of storage, and after 21 days, the milk with ascorbic acid was the lightest. Increasing the storage time to 21 days resulted in lower L* values in K, DR and AC milk. Similar to the control sample, VC milk with ascorbic acid showed a proportion of blue color and a smaller proportion of red color than DR and AC milk. The ratio of red and yellow increased most intensively with the addition of rosehip to the milk.

The redness (a*) and yellowness (b*) of DR and AC fermented milk increased during storage, contributing to a decrease in color lightness (L*). The obtained results indicate that the addition of rosehip and acerola to fermented milk might affect the color properties of milk. However, the addition of ascorbic acid differentiated the color components of fermented milk to the minimum degree during storage. Anthocyanins found in rosehip and acerola indicate a wide range of colors depending on pH. Due to the reduction in milk pH during fermentation of DR and AC milk by *L. rhamnosus*, the a* value of milk and DR and AC increased with a more extended storage period. The increase in b* value and decrease in L* lightness of DR and AC milk during storage may be related to the formation of brown polymers due to the degradation of phenolic compounds [46]. Analysis of variance ANOVA indicates that the C and h^0^ parameters of fermented milk are only significantly affected by the source of vitamin C, whereas storage time and interaction of these two factors were not significant.

### 2.5. Parameters of Texture

Hardness is the compressive force of food or sample between the tongue and palate, as the attribute that most influences consumer choice products such as yoghurt [47,48]. Adhesiveness is a parameter that shows how much the product adheres to the probe (mouth) and elastic force; it is necessary to grind this semi-solid material until it is swallowed [49,50].

The results of the TPA test in Table 3 determine the texture properties (hardness, adhesiveness, cohesiveness, springiness) of all groups of milk fermented by *L. rhamnosus*. DR and AC milk showed higher hardness and adhesiveness values compared to control milk.

The DR milk with rosehip had the highest hardness, which may be due to the pectin polysaccharide present in organic rosehip powder. According to Ognyanov et al. [51], this polysaccharide fraction consists mainly of galacturonic acid (45.5%), galactose (5.5%) and arabinose (4.7%). Presumably, homogalacturonan, which is the primary building block of pectin in rosehip consisting of long sequences of a molecule composed of 1,4-linked α-d-galacturonic acid (GalA) interspersed with small blocks of non-methyl-esterified GalA units, causes an increase in the hardness of the tested DR milk [52].

The addition of ascorbic acid to VC milk resulted in a gel with the lowest hardness on the 1st and 21st days of storage.

These results agreed with Sah et al. [53] and Tudorica et al. [54], who observed lower hardness or consistency of yoghurt with selected additives. The lower hardness could be related to structural changes in the acid gel with loss of firmness of the protein matrix [55,56,57]. The addition of vitamin C from different sources induced a change in the acid gel structure. Furthermore, Sah et al. [53] found that the decrease in firmness values in yoghurt with pineapple fiber powder could be attributed to incompatibility between milk proteins and fruit fiber polysaccharides, which was not confirmed by our study as the addition of wild rose and acerola increased hardness.

Cohesiveness measures the degree of compression to which a sample is compressed between teeth before breaking, which is the resistance of the internal connections [58]. In the present study, cohesiveness values ranged from 0.44 to 0.63. As for springiness, there was no significant effect of added vitamin C from different sources or storage time on these texture components.

### 2.6. Microbiology Analysis

**Figure 1:**
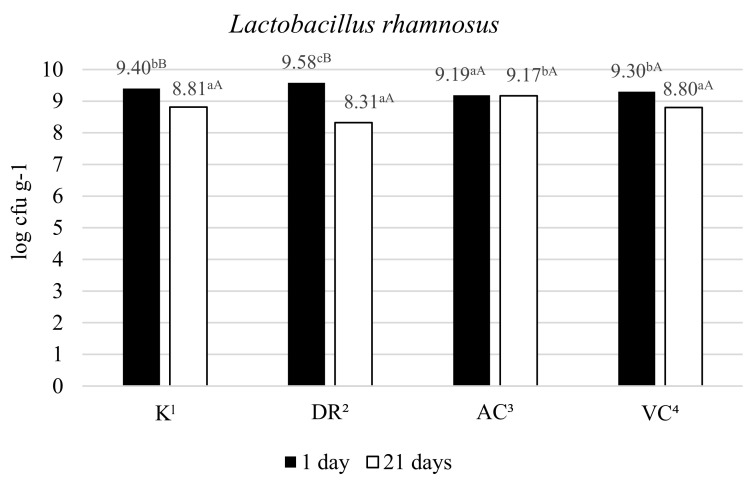
Viable counts in milk fermented by *L. rhamnosus* depending on the source of vitamin C [log cfu g^−1^]; Values are means ± SD; n = 15 for each group; ^1^—control milk; ^2^—fermented milk with rosehip; ^3^—fermented milk with acerola; ^4^ —fermented milk with L(+)ascorbic acid; A,B—mean values between 1 and 21 days of storage time denoted by different letters differ statistically significantly (*p* < 0.05); a–c—mean values between the form of vitamin C denoted by different letters differ statistically significantly (*p* < 0.05).

The viable cell counts of *Lactobacillus rhamnosus* on the 1st and 21st day of storage are shown in Figure 1. The growth of *L. rhamnosus* bacteria was enhanced by adding rosehip to the milk before fermentation. After the 1st day of storage of DR milk, the viable cell count of *L. rhamnosus* bacteria was 0.18 log cfu g^−1^ higher than the control. In the other milk samples with added vitamin C (AC and VC), the number of viable *L. rhamnosus* bacterial cells was lower compared to the control, but the differences were not significant. After the first day of storage, bacterial cell counts above 9 log cfu g^−1^ were determined in all fermented milk samples. The results of Choi and Lim [59] suggest that additional enrichment with 0.025% ascorbic acid and 0.5% yeast extract of yoghurt supplemented with 0.5% EBG (enzyme-bioconverted ginseng) may be beneficial to produce probiotic yoghurt with high amounts of *L. acidophilus* and *Bifidobacterium*. In our study, only the addition of rosehip had a stimulating effect on the growth of *L. rhamnosus*. In contrast, the addition of acerola and ascorbic acid caused a decrease in the number of bacterial cells compared to the control after one day of storage.

The number of *L. rhamnosus* cells during storage was reduced by extending the storage time to 21 days (Figure 1), especially in DR milk (by 1.2 log cfu g^−^^1^), VC milk (by 0.5 log cfu g^−^^1^) and control milk by (0.6 log cfu g^−^^1^). Only in AC fermented milk, a similar number of *L. rhamnosus* cells was determined after 21 days compared to the first day. This indicates that acerola’s addition helped increase bacterial cell viability and maintain a constant level of viable bacterial cells throughout the storage period. The lowest viability of *L. rhamnosus* cells was determined in milk with the addition of rosehip DR. These differences are most probably caused by the presence of different proportions of compounds such as amino acids, phenolic compounds, including anthocyanins and flavonoids, as well as carotenoids and vitamins [45].

The highest pH characterized DR milk at both 1 and 21 days of storage compared to the other milk. According to Collins et al. [60], *L. rhamnosus* requires for growth a high level of vitamins including folic acid, riboflavin, niacin, pantothenic acid and the mineral calcium. The optimum initial pH value for growth is in the range of 6.4 to 4.5. Therefore, *L. rhamnosus* in milk with the addition of rosehip provided good conditions for growth. It could be concluded that the decrease in the number of viable *L. rhamnosus* cells could be related to the highest pH after 21 days of storage compared to the other samples. Most probably, this pH value of DR milk was affected by the compounds present in the rosehip.

Ascorbic acid addition to fermented milk might provide a deoxygenating function and help reduce the oxidation-reduction potential necessary for the viability of probiotic bacteria [61]. In a study by Dave and Shah [38], the addition of ascorbic acid to fermented milk reduced its oxygen content and redox potential during 15 and 20 days of storage, resulting in a slower reduction of Lactobacillus cells.

On the 21st day of storage, the number of *L. rhamnosus* cells in control milk and milk with added vitamin C: DR, AC and VC reached >8 log cfu g^−^^1^; thus, these milks met the therapeutic minimum criterion (International Dairy Federation’s Recommendation) [62] for probiotic products, which should contain at least 7 log cfu g^−^^1^ of lactic acid bacteria.

### 2.7. Organoleptic Evaluation

**Figure 2:**
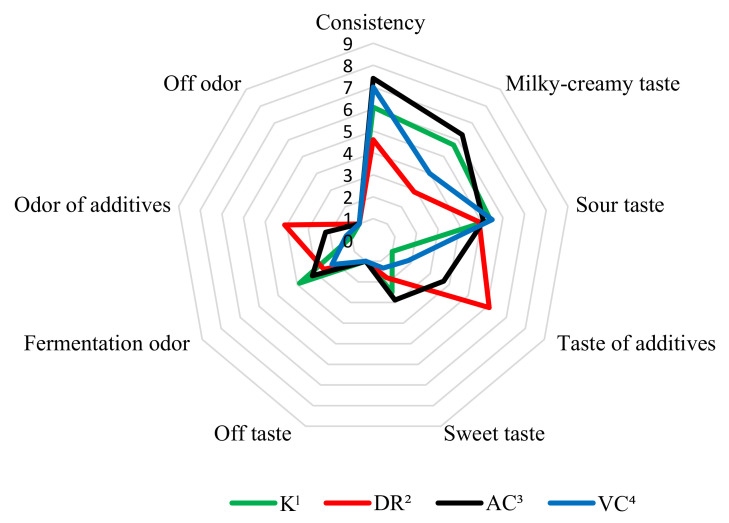
Effect of added different source of vitamin C on organoleptic parameters of fermented milk by *L. rhamnosus* after 1st day of storage; ^1^—control milk; ^2^—fermented milk with rosehip; ^3^—fermented milk with acerola; ^4^—fermented milk with L(+)ascorbic acid.

**Figure 3:**
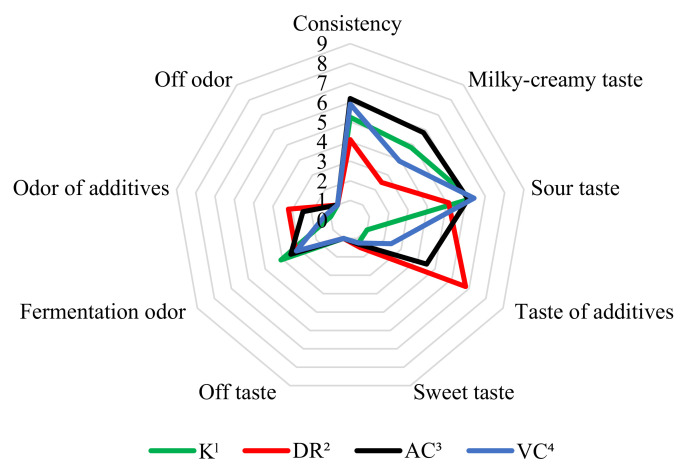
Effect of added different source of vitamin C on organoleptic parameters of fermented milk by *L. rhamnosus* after 21st day of storage; ^1^—control milk; ^2^—fermented milk with rosehip; ^3^—fermented milk with acerola; ^4^—fermented milk with L(+)ascorbic acid.

The results of the organoleptic evaluation are shown in Figure 2 and Figure 3. It was found that the addition of vitamin C from rosehips and acerola improved the consistency of fermented milk and decreased the intensity of sour taste in both evaluation dates compared to control milk. The addition of rosehip and acerola provided the fermented milks with a characteristic additive taste and aroma. According to the assessors, the taste and odor given by the addition of rosehip were the most intense of all the vitamin C sources used in the experiments. Milk with the addition of ascorbic acid VC was characterized by the most intense sour taste and a slightly perceptible smell of fermentation. The K, DR, AC, VC milks did not show any off flavor or off odor in both periods, which was confirmed by the analysis of variance (Table 2).

## 3. Material and Methods

### 3.1. Materials

Fermented milk was produced from Łaciate milk (2% fat) from SM Mlekpol (Grajewo, Poland). Organic powder of rosehips (Rosa canina L.) (400 mg of vitamin C × 100 g^−1^) came from RADZIOWI SP. z.o.o. (Częstochowa, Poland). Acerola in powder form (25,000 mg vitamin C × 100 g^−1^) was purchased from Aliness Health’n’beauty (Karczew, Poland) and L(+) ascorbic acid from Chempur (Piekary Śląskie, Poland). The starter culture of probiotic bacteria (*Lactobacillus rhamnosus*) was purchased from Chr. Hansen (Hoersholm, Denmark). MRS agars and peptone water came from Biocorp (Warszawa, Poland). Sodium hydroxide, phenolphthalein, ascorbic acid, sodium bicarbonate, oxalic acid, 2,6-dichlorophenolindophenol, were purchased from Chempur (Piekary Śląskie, Poland).

All of the reagents used were of analytical reagent grade.

#### Preliminary Studies

The amount of vitamin C addition depending on the source of origin was determined based on the preliminary experiments. The addition of higher doses of vitamin C depending on the source, to milk at 37 °C before fermentation caused denaturation of milk proteins, which also made further studies impossible. The applied amount of vitamin C was the maximum possible dose that did not cause milk protein denaturation.

### 3.2. Preparation of Fermented Milk

The milk was pasteurized at 85 °C, 30 min, then cooled to 40 °C and divided into groups:

K—control milk;
AC—fermented milk with acerola;
DR—fermented milk with rosehip;
VC—fermented milk with L(+) ascorbic acid.

Next, the milk was inoculated with a single starter culture of Lactobacillus rhamsosus (Chr. Hansen, Hvidovre, Denmark). The control sample was the milk without the addition of vitamin C. Each batch of milk was inoculated with a previously activated starter culture *Lactobacillus rhamnosus* (in the form of bulk activated at 40 °C for 5 h, which was added to the milk in the amount of 5%), according to the Szajnar et al. [63] method. Inoculated milk was stirred and poured into 100 mL plastic cups and fermented at 37 °C for 10 h. The final products were cooled to 5 °C (Cooled Incubator ILW 115, POL-EKO Aparatura, Wodzisław Śląski, Poland). The experiment was repeated three times. Fermented milk was evaluated after the first and twenty-one days of cold storage (5 °C).

### 3.3. Determination of Acidity

The pH value was performed with a pH-meter (FiveEasy Mettler Toledo, Greifensee, Switzerland) using an electrode InLab^®^Solids Pro-ISM (Mettler Toledo, Greifensee, Switzerland).

The milk’s total acidity (TA) (g of lactic acid L^−1^) was determined according to the method Jemaa et al. [64].

### 3.4. Microbiological Analysis

Each batch of milk was inoculated with a previously activated starter culture *Lactobacillus rhamnosus* (in the form of bulk activated at 40 °C for 5 h, which was added to the milk in the amount of 5%), according to the Lima et al. [65] method. Inoculated milk was stirred and poured into 100 mL plastic cups and fermented at 37 °C for 10 h.

### 3.5. Syneresis

Syneresis was measured as the amount of whey released relative to the initial weight and averaged five determinations. The 10 g of fermented milk sample was transferred into 50 mL plastic tube and centrifuged Refrigerated Centrifuge LMC-4200R (Biosan SIA, Riga, Latvia) [66].

### 3.6. Color of Fermented Milk

The color of fermented milk was analyzed with a colorimeter (the Precision Colorimeter, Model NR 145, Shenzhen, China) using the instrumental method and the CIELAB system. The image lightness was determined with the parameter L* and chromaticity using a*, b*, C, h^0^. Prior to the measurement, the device was calibrated on a white reference standard [67].

### 3.7. Determination of Vitamin C Content

The content of ascorbic acid was determined in the samples by Tillimans’ titration method [PN-A-04019:1998] [68], which is based on the extraction of ascorbic acid from the product with oxalic acid, followed by its oxidation to dehydroascorbic acid in an acidic medium using the titrated blue dye 2,6-dichlorophenolindophenol (DCIP).

### 3.8. Parameters of Texture

Texture profile was determined with TPA test using CT3 Texture Analyzer (Brookfield, Middleboro, MA, USA) with Texture Pro CT (Brookfield, Middleboro, MA, USA) software. The dimensions of the sample were: 66 mm × 33.86 mm (cylinder), and the sample temperature was 8 °C. The experiment was conducted using the acrylic probe TA 3/100 with the following settings: distance 15 mm, contact load 0.1 N, measurement speed 1 mm/s [67].

### 3.9. Organoleptic Evaluation

The analysis was made for three fermented milk samples with vitamin C and the control sample. The organoleptic evaluation was performed by a trained panel (15 women and 15 men, the age of 25–30). The panelists were served four samples at a time and asked to rinse their mouths with water between samples. The samples of fermented milk were assessed on a 9-point rating scale with edge markings. The left end denoted the least intense, the least characteristic feature. The panelists evaluated the presence of sandy and grainy consistency; impalpable milky-creamy taste, impalpable sour taste, impalpable fermentation odor, impalpable sweet taste, impalpable the off taste and odor, impalpable additives taste and odor, and the right end denoted the most characteristic feature: smooth texture; the most intense milky-creamy taste, the most intense sour taste, the most intense fermentation odor, the most intense sweet taste, the most intense the off taste and odor, the most intense characteristic taste and odor of additives [69], PN-ISO 22935-2:2013-07 [70].

Definition of the attributes in the descriptive organoleptic analysis of fermented milk:

Milky-creamy taste: the taste stimulated by milk powder.
Sour taste: the taste stimulated by lactic acid.
Taste of additives: the taste stimulated by added vitamin C depending on the source of origin.
Sweet taste: the taste stimulated by sucrose.
Off-taste: an unidentified taste that is not characteristic.
Fermentation odor: the intensity of odor associated with sour milk, i.e., lactic acid.
Odor of additives: odor characteristic stimulated by added vitamin C depending on the source of origin.
Off-odor: unidentified odor that is not characteristic.

### 3.10. Statistical Analysis

The results from three independent studies were expressed as the mean and standard deviation in Statistica v. 13.1 (StatSoft, Tulsa, OK, USA). One and two-way ANOVA was performed, and the differences between the mean values were verified with the Turkey test, with *p* < 0.05.

## 4. Conclusions

This study showed good stability of vitamin C from different sources (rosehip, acerola and ascorbic acid in powder form) in fermented milk, as losses were only 5–8%. Depending on the vitamin C source, the color, hardness and organoleptic characteristics of the probiotic milk were significantly affected. After 21 days of storage, all tested milks contained >8 log cfu g^−1^ *L. rhamnosus*, which provided a therapeutic effect. Due to the advantages of natural products and favorable consumer perception, the addition of rosehip and acerola could be considered promising candidates for developing the functional food market.

## Figures and Tables

**Table 1:** Vitamin C, color, syneresis, pH and total acidity of fermented milk.

Properties		Storage Time	K ^1^	DR ^2^	AC ^3^	VC ^4^
pH		0	6.65 ^cB^ ± 0.03	5.33 ^aC^ ± 0.11	6.52 ^bC^ ± 0.06	6.26 ^dC^ ± 0.02
		1	4.65 ^bA^ ± 0.30	4.86 ^cB^ ± 0.02	4.38 ^aB^ ± 0.01	4.76 ^bB^ ± 0.05
		21	4.39 ^aA^ ± 0.09	4.70 ^cA^ ± 0.02	4.30 ^aA^ ± 0.01	4.42 ^bA^± 0.01
Total acidity, g lactic acid L^−1^		1	0.72 ^bA^ ± 0.02	0.69 ^bA^ ± 0.01	0.83 ^cA^ ± 0.05	0.65 ^aA^ ± 0.04
		21	0.74 ^aA^ ± 0.14	0.70 ^aA^ ± 0.01	0.75 ^aA^ ± 0.07	0.72 ^aA^ ± 0.04
Vitamin C, mg 100 g^−1^		0	10.58 ^aC^ ± 0.36	43.33 ^bC^ ± 0.20	43.06 ^bC^ ± 0.30	43.72 ^bB^ ± 0.30
		1	9.52 ^aB^ ± 0.26	42.30 ^bB^ ± 0.20	42.00 ^bB^ ± 0.10	43.06 ^cB^ ± 0.39
		21	5.10 ^aA^ ± 0.51	40.72 ^bA^ ± 0.11	40.30 ^bA^ ± 0.21	40.22 ^bA^ ± 0.10
Syneresis, %		1	64.23 ^bA^ ± 1.18	61.93 ^aA^ ± 1.05	72.53 ^cB^ ± 0.59	61.37 ^aA^ ± 1.29
		21	65.86 ^aA^ ± 0.66	60.20 ^cA^ ± 0.38	64.05 ^bA^ ± 0.34	63.19 ^bB^ ± 0.96
Color	L*	1	94.96 ^cA^ ± 1.85	58.02 ^aB^ ± 1.66	86.95 ^bB^ ± 1.21	95.04 ^cA^ ± 1.46
		21	90.60 ^aA^ ± 4.26	51.42 ^aA^ ± 2.52	82.38 ^bA^ ± 0.22	94.20 ^dA^ ± 0.42
	a*	1	−1.81 ^aA^ ± 0.29	3.88 ^cA^ ± 0.30	1.54 ^bA^ ± 0.25	−1.55 ^aB^ ± 0.18
		21	−1.89 ^aA^ ± 0.35	6.37 ^dB^ ± 0.27	1.69 ^cA^ ± 0.33	−1.32 ^bA^ ± 0.02
	b*	1	8.54 ^aA^ ± 0.97	17.74 ^cA^ ± 1.21	9.06 ^bA^ ± 0.55	6.89 ^aA^ ± 0.47
		21	8.12 ^bA^ ± 0.21	19.24 ^dA^ ± 0.74	10.20 ^cA^ ± 0.89	6.84 ^aA^ ± 0.12
	C	1	8.76 ^aA^ ± 1.98	18.16 ^bA^ ± 1.21	9.19 ^aA^ ± 1.57	7.06 ^aA^ ± 0.47
		21	8.39 ^bA^ ± 0.24	20.56 ^dB^ ± 0.04	10.35 ^cA^ ± 0.91	6.96 ^aA^ ± 0.11
	h^0^	1	102.30 ^cA^ ± 1.34	77.67 ^aB^ ± 0.31	99.72 ^bA^ ± 0.42	102.72 ^cA^ ± 1.52
		21	103.37 ^cA^ ± 0.28	72.07 ^aA^ ± 0.99	99.42 ^bA^ ± 0.88	100.92 ^bA^ ± 0.29

^1^—control milk; ^2^—fermented milk with rosehip; ^3^—fermented milk with acerola; ^4^—fermented milk with l(+)ascorbic acid; 0—before fermentation; 1—after 1st day of storage; 21—after 21st day of storage; Values are mean ± SD; n = 15 for each group; A,B—mean values in columns denoted by different letters differ statistically significantly (*p* ˂ 0.05) depending on the storage time; a–c—mean values in lines denoted by different letters differ statistically significantly (*p* ˂ 0.05) depending on the different forms of vitamin C. Color: L* as brightness; a* color from red (+) to green (-); b* as the colors from yellow (+) to blue (-); C as the purity and intensity of the color, h⁰ as the shade of the color.

**Table 2:** Analysis of variance (ANOVA) *p*-values on the effects of storage time and the source of vitamin C addition on vitamin C, pH, total acidity, syneresis, color parameters: L*, a*, b*, C, h^0^, hardness, adhesiveness, stringiness length, cohesiveness, springiness, preference, consistency, milky-creamy taste, sour taste, taste of additives, sweet taste, off taste, sour odor, odor of additives, off odor, *L. rhamnosus* of fermented milk.

Properties	Storage Time *p*-Values	Source of Vitamin C *p*-Values	Storage Time × Source of Vitamin C *p*-Values
Vitamin C	0.0018↑	0.1010 n.s.	0.0510 n.s.
pH	0.0003↑	0.0003↑	0.0138↑
Total acidity	0.9937 n.s.	0.0139↑	0.5691 n.s.
Syneresis	0.0073↑	0.0262↑	0.0065↑
L*	0.0907↑	0.0000↑	0.0091↑
a*	0.0000↑	0.0000↑	0.0000↑
b*	0.0907 n.s.	0.0000↑	0.0863 n.s.
C	0.0719 n.s.	0.0000↑	0.0482
h^0^	0.0503 n.s.	0.0000↑	0.0512 n.s.
Hardness	0.7351 n.s.	0.0000↑	0.8059 n.s.
Adhesiveness	0.6535 n.s.	0.1805 n.s.	0.7476↑
Cohesiveness	0.3739 n.s.	0.0860 n.s.	0.9642 n.s.
Springiness	0.4008 n.s.	0.0514 n.s.	0.7199 n.s.
Consistency	0.0002↑	0.0006↑	0.0003↑
Milky-creamy taste	0.0004↑	0.0126↑	0.0261↑
Sour taste	0.0003↑	0.1947 n.s.	0.1588 n.s.
Taste of additives	0.0824 n.s.	0.0340↑	0.4515 n.s.
Sweet taste	0.0321↑	0.0451↑	0.0472↑
Off-taste	0.8451 n.s.	0.7142 n.s.	0.9411 n.s.
Fermentation odor	0.0295↑	0.0140↑	0.0092↑
Odor of additives	0.7419 n.s	0.0121↑	0.8134 n.s.
Off-odor	0.6912 n.s.	0.4120 n.s.	0.0529 n.s.
*L. rhamnosus*	0.0228↑	0.0362↑	0.0014↑

Storage time × Source of vitamin C = interaction; ↑ indicates significant effect p ≤ 0.05; n.s.: no significant effect.

**Table 3:** Parameters of texture profile of milk fermented by *Lactobacillus rhamnosus* depending on the source of vitamin C.

Properties	Storage Time	K ^1^	DR ^2^	AC ^3^	VC ^4^
Hardness, N	1	0.91 ^aA^ ± 0.52	2.29 ^bA^ ± 0.18	1.37 ^bA^ ± 0.20	0.78 ^aA^ ± 0.05
	21	0.92 ^aA^ ± 0.45	1.93 ^bA^ ± 0.27	1.51 ^bA^ ± 0.04	0.78 ^aA^ ± 0.13
Adhesiveness, mJ	1	1.10 ^aA^ ± 0.95	1.73 ^bB^ ± 0.19	1.77 ^bA^ ± 0.21	1.70 ^bB^ ± 0.26
	21	1.04 ^bA^ ± 0.17	1.13 ^bA^ ± 0.13	1.83 ^cA^ ± 0.05	0.67 ^aA^ ± 0.06
Cohesiveness	1	0.63 ^aA^ ± 0.19	0.55 ^aA^ ± 0.21	0.46 ^aA^ ± 0.02	0.63 ^aA^ ± 0.22
	21	0.56 ^aA^ ± 0.09	0.50 ^aA^ ± 0.07	0.44 ^aA^ ± 0.12	0.59 ^aA^ ± 0.06
Springiness, mm	1	13.64 ^aA^ ± 0.54	14.74 ^aA^ ± 0.67	13.45 ^aA^ ± 0.27	13.94 ^aA^ ± 0.53
	21	13.41 ^aA^ ± 0.75	14.02 ^aA^ ± 0.56	13.55^aA^ ± 0.35	13.93^aA^ ± 0.14

^1^—control milk; ^2^—fermented milk with rosehip; ^3^—fermented milk with acerola; ^4^—fermented milk with L(+)ascorbic acid; 1—after 1st day of storage; 21—after 21st day of storage; Values are mean ± SD; n = 15 for each group; A,B—mean values in columns denoted by different letters differ statistically significantly (*p* ˂ 0.05) depending on the storage time; a–c—mean values in lines denoted by different letters differ statistically significantly (*p* ˂ 0.05) depending on the different forms of vitamin C.

## Data Availability

Not applicable.

## Sample Availability

Samples of the compounds are not available from the authors.

## References

[1] Cremonini F., Di Caro S., Nista E.C., Bartolozzi F., Capelli G., Gasbarrini G., Gasbarrini A. (2002). Meta-analysis: The effect of probiotic administration on antibiotic-associated diarrhea. Aliment. Pharmacol. Ther..

[2] Johnston B.C., Supina A.L., Ospina M., Vohra S. (2007). Probiotics for the prevention of pediatric antibotic-associated diarrhea. Cochrane Database Syst. Rev..

[3] Lee J., Seto D., Bielory L. (2008). Meta-analysis of clinical trials of probiotics for prevention and treatment of pedatric atopic dermatitis. J. Allergy Clin. Immunol..

[4] Oleksy-Sobczak M., Klewicka E., Piekarska-Radzik L. (2020). Exopolysaccharides production by *Lactobacillus rhamnosus* strains–Optimization of synthesis and extraction conditions. LWT-Food Sci. Technol..

[5] Sharareh H., Soltani H., Reid G. (2009). Growth and survival of *Lactobacillus* reuteri RC-14 and *Lactobacillus rhamnosus* GR-1 in yogurt for use as a functional food. Innov. Food Sci. Emerg. Technol..

[6] Jyoti B.D., Suresh A.K., Venkatesh K.V. (2004). Effect of preculturing conditions on growth of *Lactobacillus rhamnosus*, on medium containing glucose and citrate. Microbiol. Res..

[7] Innocente N., Biasutti M., Rita F., Brichese R., Comi G., Iacumin L. (2016). Effect of indigenous *Lactobacillus rhamnosus* isolated from bovine milk on microbiological characteristics and aromatic profile of traditional yogurt, *Lebensm*. Wiss. Technol..

[8] Kamal R.M., Alnakip M.E., Abd El Aal S.F., Bayoumi M.A. (2018). Bio-controlling capability of probiotic strain *Lactobacillus rhamnosus* against some common foodborne pathogens in yoghurt. Int. Dairy J..

[9] Maćkowiak K., Torliński L. (2007). Współczesne poglądy na rolę witaminy C w filozjologii i patologii człowieka. Contemporary view on the role of vitamin c in human physiology and pathology. Now. Lek..

[10] Miktus M. (2000). Witaminy część II: Ogólna charakterystyka witaminy C. Vitamins part II: General characteristics of vitamin C. Zyw. i Zdrowie..

[11] Kleszczewska E. (2007). Biologiczne znaczenie witaminy C ze szczególnym uwzględnieniem jej znaczenia w metabolizmie skóry. Biological role of vitamin C and importance in the skin metabolism. Pol. Merkur. Lek..

[12] Zhang P.Y., Xu X., Li X.C. (2014). Cardiovascular diseases: Oxidative damage and antioxidant protection. Eur. Rev. Med. Pharmacol. Sci..

[13] Prakash A., Baskaran R. (2018). Acerola, an untapped functional superfruit: A review on latest frontiers. J. Food Sci. Technol..

[14] Marques T.R., Caetano A.A., Simão A.A., Castro F.C.D.O., Ramos V.D.O., Corrêa A.D. (2016). Methanolic extract of Malpighia emarginata bagasse: Phenolic compounds and inhibitory potential on digestive enzymes. Rev. Bras. Farmacogn..

[15] Hanamura T., Uchida E., Aoki H. (2008). Changes of the composition in acerola (Malpighia emarginata DC.) fruit in relation to cultivar, growing region and maturity. J. Sci. Food Agric..

[16] Hanamura T., Uchida E., Aoki H. (2008). Skin-lightening effect of a polyphenol extract from Acerola (Malpighia emarginata DC.) fruit on UV-induced pigmentation. Biosci. Biotechnol. Biochem..

[17] Righetto A.M., Netto F.M., Carraro F. (2005). Chemical composition and antioxidant activity of juices from mature and immature acerola (Malpighia emarginata DC). Food Sci. Technol. Int..

[18] Vendramini A.L., Trugo L.C. (2000). Chemical composition of acerola fruit (Malpighia punicifolia L.) at three stages of maturity. Food Chem..

[19] Mármol I., Sánchez-de-Diego C., Jiménez-Moreno N., Ancín-Azpilicueta C., Rodríguez-Yoldi M.J. (2017). Therapeutic Applications of Rose Hips from Different Rosa Species. Int. J. Mol. Sci..

[20] Hvattum E. (2002). Determination of phenolic compounds in rose hip (Rosa canina) using liquid chromatography soupled to electrospray ionization tandem mass spectrometry and diode-array detection. Rapid Commun. Mass Spetrometry.

[21] Adamczak A., Buchwald W., Zieliński J., Mielcarek S. (2012). Flavonoid and organic acid content in rose hips (Rosa, L., sect. Caninae DC. EM. Christ.). Acta Biol. Crac. Ser. Bot..

[22] Nojavan S., Khalilian F., Kiaie F.M., Rahimi A., Arabanian A., Chalavi S. (2008). Extraction and quantitative determination of ascorbic acid during different maturity stages of Rosa canina L. fruit. J. Food Compos. Anal..

[23] Ball G.F.M. (2006). Vitamins in Foods/Analysis, Bioavailability, and Stability.

[24] Linares D., Michaud P., Delort A.M., Traïkia M., Warrand J. (2011). Catabolism of L-ascorbate by *Lactobacillus rhamnosus* GG. J. Agric. Food Chem..

[25] Pérez-Vicente A., Gil-Izquierdo A., García-Viguera C. (2002). In vitro gastrointestinal digestion study of pomegranate juice phenolic compounds, anthocyanins, and vitamin C. J. Agric. Food Chem..

[26] Yuan J.P., Chen F. (1998). Degradation of ascorbic acid in aqueous solution. J. Agric. Food Chem..

[27] Budsławski J. (1971). Zarys chemii mleka. Outline of milk chemistry Państwowe Wydawnictwo Rolnicze i Leśne.

[28] Klupsch H.J., Verlag T. (1992). Acid Milk Products, Milk Beverage and Desserts.

[29] Ogah O., Watkins C.S., Ubi B.E., Oraguzie N.C. (2014). Phenolic compounds in Rosaceae fruit and nut crops. J. Agric. Food Chem..

[30] Demir N., Yildiz O., Alpaslan M., Hayaloglu A. (2014). Evaluation of volatiles, phenolic compounds and antioxidant activities of rose hip (Rosa L.) fruits in Turkey. LWT-Food Sci. Technol..

[31] Elmastas M., Demir A., Genc N., Dölek Ü., Günes M. (2017). Changes in flavonoid and phenolic acid contents in some Rosa species during ripening. Food Chem..

[32] Nadpal J.D., Lesjak M.M., Šibul F.S., Anackov G.T., Cetojevic’-Simin D.D., Mimica-Dukic N.M., Beara I.N. (2016). Comparative study of biological activities and phytochemical composition of two rose hips and their preserves: Rosa canina L. and Rosa arvensis Huds. Food Chem..

[33] Tumbas V.T., Canadanovic-Brunet J.M., Cetojevic-Simin D.D., Cetkovic G.S., Ethilas S.M., Gille L. (2012). Effect of rosehip (Rosa canina L.) phytochemicals on stable free radicals and human cancer cells. J. Sci. Food Agric..

[34] Hosni K., Chrif R., Zahed N., Abid I., Medfei W., Sebei H., Brahim N.B. (2010). Fatty acid and phenolic constituents of leaves, flowers and fruits of tunisian dog rose (Rosa canina L.). Riv. Ital. Sostanze Grasse.

[35] Türkben C., Uylaşer V., İncedayı B., Çelikkol I. (2010). Effects of different maturity periods and processes on nutritional components of rose hip (Rosa canina L.). J. Food Agric. Environ..

[36] Olsson M.E., Gustavsson K.E., Andersson S., Nilsson A., Duan R.D. (2004). Inhibition of cancer cell proliferation in vitro by fruit and berry extracts and correlations with antioxidant levels. J. Agric. Food Chem..

[37] Ilic D.B., Ashoor S.H. (1988). Stability of Vitamins A and C in Fortified Yogurt. J. Dairy Sci..

[38] Dave R.I., Shah N.P. (1997). Effectiveness of ascorbic acid as an oxygen scavenger in improving viability of probiotic bacteria in yoghurts made with commercial starter cultures. Int. Dairy J..

[39] Graulet B. (2014). Ruminant milk: A source of vitamins in human nutrition. Anim. Front..

[40] Rani R., Dharaiya C.N., Unnikrishnan V., Singh B. (2012). Factors Affecting Syneresis from Yoghurt for Preparation of Chakka. Indian J. Dairy Sci..

[41] Peng Y., Horne D.S., Lucey J.A. (2009). Impact of preacidification of milk and fermentation time on the properties of yogurt. J. Dairy Sci..

[42] Oktavia H., Radiati L.E., Rosyidi D. (2016). Evaluation of Physicochemical Properties and Exopolysaccharides Production of Single Culture and Mixed Culture in Set Yoghurt. J-PAL.

[43] Sun J., Chen H., Qiao Y., Liu G., Leng C., Zhang Y., Lv X., Feng Z. (2019). The nutrient requirements of *Lactobacillus rhamnosus* GG and their application to fermented milk. J. Dairy Sci..

[44] Kaszuba M., Viapiana A., Wesołowski M. (2019). Dzika róża (Rosa canina L.) jako źródło witamin i przeciwutleniaczy w diecie człowieka. Rose hip (Rosa canina L.) as a vitamin and antioxidants source in human diet. Pol. Tow. Farm..

[45] Belwal T., Devkota H.P., Hassan H.A., Ahluwalia S., Ramadan M.F., Mocan A., Atanasov A.G. (2018). Phytopharmacology of Acerola (Malpighia spp.) and its potential as functional food. Trends Food Sci. Technol..

[46] Lin D., Xiao M., Zhao J., Li Z., Xing B., Li X., Kong M., Li L., Zhang Q., Liu Y. (2016). An Overview of Plant Phenolic Compounds and Their Importance in Human Nutrition and Management of Type 2 Diabetes. Molecules.

[47] Damin M.R., Alcântara M.R., Nunes A.P., Oliveira M.N. (2009). Effects of milk supplementation with skim milk powder, whey protein concentrate and sodium caseinate on acidification kinetics, rheological properties and structure of nonfat stirred yogurt. LWT-Food Sci. Technol..

[48] Macit E., Bakirci İ. (2017). Effect of different stablizers on quality characteristics of the set-type yoğurt. Afr. J. Biotechnol..

[49] Bourne M. (2002). Food Texture and Viscosity Concept and Measurement.

[50] Mousavi M., Heshmati A., Daraei Garmakhany A., Vahidinia A., Taheri M. (2019). Texture and sensory characterization of functional yogurt supplemented with flaxseed during cold storage. Food Sci. Nutr..

[51] Ognyanova M., Remoroza C., Schols H.A., Georgiev Y., Kratchanova M., Kratchanov C. (2016). Isolation and structure elucidation of pectic polysaccharide from rose hip fruits (Rosa canina L.). Carbohydr. Polym..

[52] Albersheim P., Darvill A.G., O’Neill M.A., Schols H.A., Voragen A.G.J., Visser J., Voragen A.G.J. (1996). An Hypothesis: The Same Six Polysaccharides are Components of the Primary Cell Walls of All Higher Plants.

[53] Sah B.N.P., Vasiljevic T., McKechnie S., Donkor O.N. (2016). Physicochemical, textural and rheological properties of probiotic yogurt fortified with fibre-rich pineapple peel powder during refrigerated storage. LWT-Food Sci. Technol..

[54] Tudorica C.M., Jones T.E.R., Kuri V., Brennan C.S. (2004). The effects of refined barley beta-glucan on the physico-structural properties of low-fat dairy products: Curd yield, microstructure, texture and rheology. J. Sci. Food Agric..

[55] Lee W.J., Lucey J.A. (2010). Formation and physical properties of yogurt. Asian-Australas. J. Anim. Sci..

[56] Santillan-Urquiza E., Mendez-Rojas M.A., Velez-Ruiz J.F. (2017). Fortification of yogurt with nano and micro sized calcium, iron and zinc, effect on the physicochemical and rheological properties. LWT-Food Sci. Technol..

[57] Tan P.Y., Tan T.B., Chang H.W., Tey B.T., Chan E.S., Lai O.M., Baharin B.S., Nehdi I.A., Tan C.P. (2018). Effects of storage and yogurt matrix on the stability of tocotrienols encapsulated in chitosan-alginate microcapsules. Food Chem..

[58] Curti C.A., Vidal P.M., Curti R.N., Ramon A.N. (2017). Chemical characterization, texture and consumer acceptability of yogurts supplemented with quinoa flour. Food Sci. Technol..

[59] Choi S.H., Lim Y.S. (2019). Viability of Probiotic Bacteria in Yogurt Supplemented with Enzyme-Bioconverted Ginseng, Ascorbic Acid, and Yeast Extract. J. Dairy Sci. Biotechnol..

[60] Collins F.L., Rios-Arce N.D., Schepper J.D., Parameswaran N., McCabe L.R. (2017). The Potential of Probiotics as a Therapy for Osteoporosis. Microbiol. Spectr..

[61] Talwalkar A., Kailasapathy K. (2004). A review of oxygen toxicity in probiotic yogurts: Influence on the survival of probiotic bacteria and protective techniques. Compr. Rev. Food Sci. Food Saf..

[62] Champagne C.P., . Gardner N.J., Roy D. (2005). Challenges in the Addition of Probiotic Cultures to Foods. Crit. Rev. Food Sci. Nutr..

[63] Szajnar K., Znamirowska A., Kuźniar P. (2020). Sensory and textural properties of fermented milk with viability of *Lactobacillus rhamnosus* and *Bifidobacterium animalis* ssp. *lactis* Bb-12 and increased calcium concentration. Int. J. Food Prop..

[64] Jemaa M.B., Falleh H., Neves M.A., Isoda H., Nakajima M., Ksouri R. (2017). Quality Preservation of Deliberately Contaminated Milk Using Thyme Free and Nanoemulsified Essential Oils. Food Chem..

[65] Lima K.G., Kruger M.F., Behrens J., Destro M.T., Landgraf M., Franco B.D.G. (2009). Evaluation of Culture Media for Enumeration of *Lactobacillus acidophilus, Lactobacillus casei* and *Bifidobacterium animalis* in the presence of *Lactobacillus delbrueckii* subsp *bulgaricus* and *Streptococcus thermophilus*. LWT-Food Sci. Technol..

[66] Szajnar K., Znamirowska A., Kalicka D. (2019). Effects of various magnesium salts for the production of milk fermented by Bifidobacterium animalis ssp. lactis Bb-12. Int. J. Food Prop..

[67] Szajnar K., Pawlos M., Znamirowska A. (2021). The Effect of the Addition of Chokeberry Fiber on the Quality of Sheep’s Milk Fermented by *Lactobacillus rhamnosus* and *Lactobacillus acidophilus*. Int. J. Food Sci..

[68] (1998). PN-A-04019:1998. Produkty Spożywcze—Oznaczanie Zawartości Witaminy C. Food Products—Determination of Vitamin C.

[69] Baryłko-Pikielna N., Matuszewska I. (2014). Sensoryczne Badania Żywności. Podstawy—Metody—Zastosowania. Sensory Food Testing. Fundamentals-Methods-Applications. Wyd. Nauk. Pttż. Krakow..

[70] (2013). PN-ISO 22935-2:2013-07. Milk and Milk Products—Sensory Analysis—Part. 2: Recommended Methods for Sensory Evaluation.

